# Evolution of Telomeres in *Schizosaccharomyces pombe* and Its Possible Relationship to the Diversification of Telomere Binding Proteins

**DOI:** 10.1371/journal.pone.0154225

**Published:** 2016-04-21

**Authors:** Regina Sepsiova, Ivona Necasova, Smaranda Willcox, Katarina Prochazkova, Peter Gorilak, Jozef Nosek, Ctirad Hofr, Jack D. Griffith, Lubomir Tomaska

**Affiliations:** 1 Department of Genetics, Comenius University in Bratislava, Faculty of Natural Sciences, Ilkovicova 6, 842 15, Bratislava, Slovak Republic; 2 Chromatin Molecular Complexes, Central European Institute of Technology, Masaryk University, Brno, CZ-62500, Czech Republic; 3 Laboratory of Functional Genomics and Proteomics, National Centre for Biomolecular Research, Faculty of Science, Masaryk University, Brno, CZ-62500, Czech Republic; 4 Lineberger Comprehensive Cancer Center, University of North Carolina at Chapel Hill, Chapel Hill, North Carolina, 27599, United States of America; 5 Department of Biochemistry, Comenius University in Bratislava, Faculty of Natural Sciences, Ilkovicova 6, 842 15, Bratislava, Slovak Republic; University of Leicester, UNITED KINGDOM

## Abstract

Telomeres of nuclear chromosomes are usually composed of an array of tandemly repeated sequences that are recognized by specific Myb domain containing DNA-binding proteins (telomere-binding proteins, TBPs). Whereas in many eukaryotes the length and sequence of the telomeric repeat is relatively conserved, telomeric sequences in various yeasts are highly variable. *Schizosaccharomyces pombe* provides an excellent model for investigation of co-evolution of telomeres and TBPs. First, telomeric repeats of *S*. *pombe* differ from the canonical mammalian type TTAGGG sequence. Second, *S*. *pombe* telomeres exhibit a high degree of intratelomeric heterogeneity. Third, *S*. *pombe* contains all types of known TBPs (Rap1p [a version unable to bind DNA], Tay1p/Teb1p, and Taz1p) that are employed by various yeast species to protect their telomeres. With the aim of reconstructing evolutionary paths leading to a separation of roles between Teb1p and Taz1p, we performed a comparative analysis of the DNA-binding properties of both proteins using combined qualitative and quantitative biochemical approaches. Visualization of DNA-protein complexes by electron microscopy revealed qualitative differences of binding of Teb1p and Taz1p to mammalian type and fission yeast telomeres. Fluorescence anisotropy analysis quantified the binding affinity of Teb1p and Taz1p to three different DNA substrates. Additionally, we carried out electrophoretic mobility shift assays using mammalian type telomeres and native substrates (telomeric repeats, histone-box sequences) as well as their mutated versions. We observed relative DNA sequence binding flexibility of Taz1p and higher binding stringency of Teb1p when both proteins were compared directly to each other. These properties may have driven replacement of Teb1p by Taz1p as the TBP in fission yeast.

## Introduction

Telomeres are specialized nucleo-protein structures protecting the ends of linear DNA chromosomes against degradation and inappropriate DNA recombinational repair thus ensuring chromosome stability [1–4]. Nuclear telomeres are composed of an array of repetitive sequences and share several basic characteristics. One common feature is that their double-stranded regions are recognized by a special class of telomere-binding proteins (TBPs) characterized by the presence of at least one Myb domain that mediates their DNA binding [4,5]. Two Myb domains are required for high-affinity binding of TBPs to telomeric DNA (e.g. [6]). When a protein (such as mammalian TRF1 and TRF2) contains only a single Myb domain then it binds to the telomeric DNA as a homodimer.

The importance of TBPs for telomere functions is underlined by the effects of their absence on the stability of chromosomal ends. For example, overexpression or downregulation of either TRF1 or TRF2 results in profound effects on telomere (and genomic) stability (reviewed in [4]). Such results indicate that during their evolution the DNA-binding properties of TBPs were finely tuned to fulfill their functions.

Although the overall organization of nuclear telomeres in ascomycetous yeasts is similar to that of other eukaryotes, individual yeast species exhibit a remarkably high degree of variability in the sequence and length of the telomeric repeats. For example, the repeat units are often very long (>20 bp compared with the 6 bp TTAGGG repeat in mammals), sometimes heterogeneous (e.g. in *Saccharomyces cerevisiae* or *Schizosaccharomyces pombe*), and not always GC-rich [7–9]. Even closely related species like *Candida parapsilosis*, *C*. *orthopsilosis* and *C*. *metapsilosis* exhibit differences in the sequences of their telomeric repeats underlining a relatively high frequency of nucleotide substitutions [7]. This extraordinary variability in the sequence of the telomeres poses an important question: how are the TBPs able to “keep up” in the evolutionary race with their cognate ligands?

There are three possible solutions to this problem, as recently suggested by Steinberg-Neifach and Lue [9]: (i) combinatorial recognition of the target site (exemplified by recognition of a complex telomere repeat sequence of *Candida parapsilosis* by heterodimers of Cdc13A and Cdc13B [10]); (ii) flexibility of the recognition surfaces of the DNA-binding proteins to adopt alternative conformations; and (iii) duplication of the recognition protein and functional specialization. The possibility (ii) is supported by studies of TBPs from a large group of ascomycetous yeasts employing Rap1p as the major TBP protein (reviewed in [9]). In contrast to its mammalian homologue containing a single Myb domain displaying a weak binding to DNA [11] (but see also [12]), Rap1p in *Saccharomyces cerevisiae* contains two DNA-binding domains (DBDs) [13,14]. Their special feature is that they exhibit a high flexibility in recognizing a relatively wide range of sequences [15–18]. Thus, divergence of telomeric repeats in this group of yeasts is allowed as long as they fit into the flexible DNA pocket of Rap1p.

**Fig 1:**
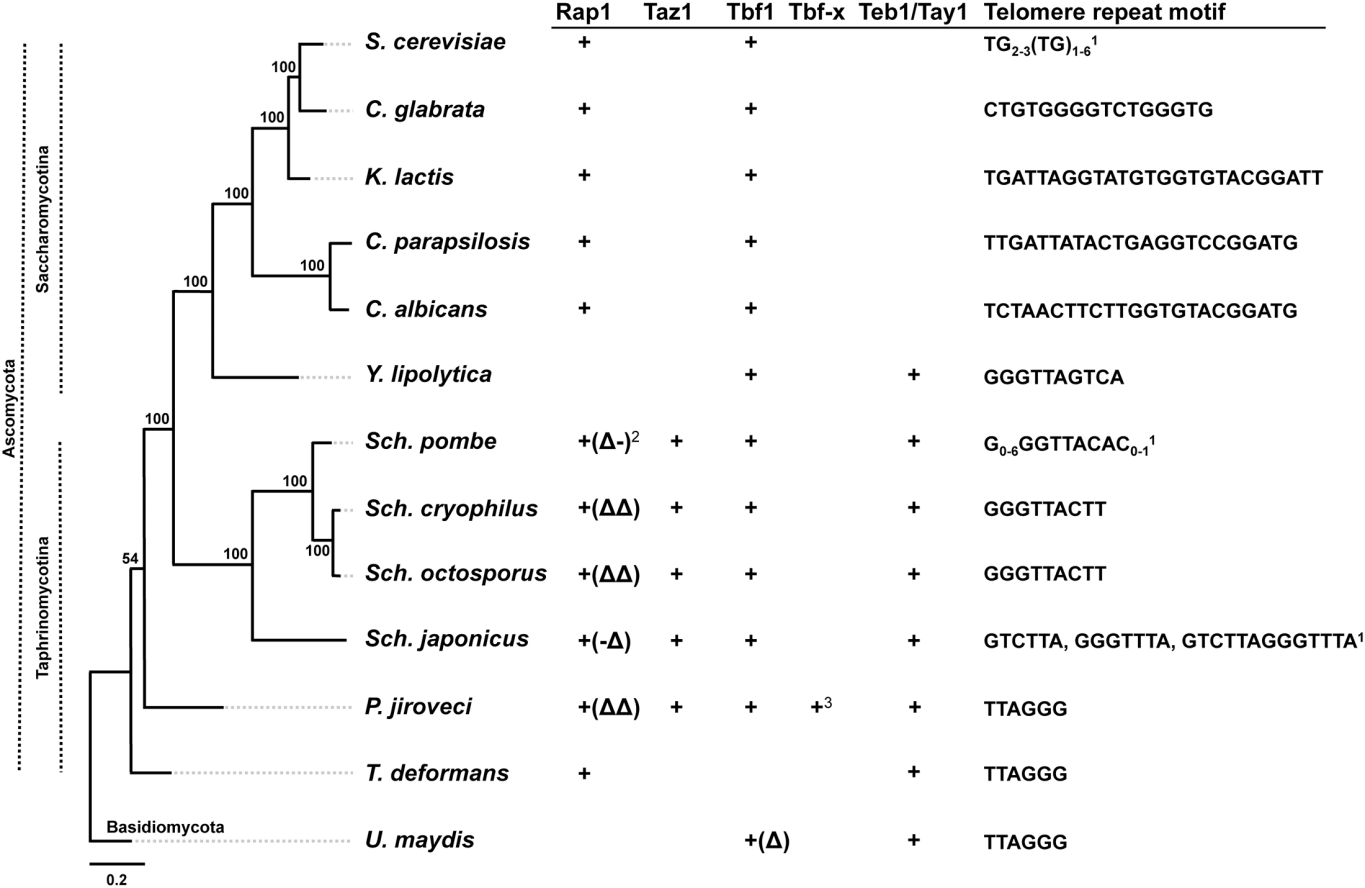
Distribution of various types of TBPs in phylogenetically distant yeast species. Whereas Tbf1p is present in most species, there is no evidence that it binds to telomeres *in vivo*. In ascomycete species the major TBP is Rap1p possessing two Myb-like DNA-binding domains (DBD) (Table 2). The exceptions are *Y*. *lipolytica* that apparently does not contain a Rap1p homologue, *S*. *pombe* Rap1p that associates with telomeres via protein-protein interactions [27] and *S*. *japonicus*, *S*. *cryophilus*, *S*. *octosporus* and *P*. *jiroveci* homologues that lack one or both DBDs. In *Y*. *lipolytica* as well as in the basidiomycete *U*. *maydis*, the TBP is represented by Tay1p [6,22]. In *S*. *pombe*, the role of Tay1p homologue (Teb1p) at telomeres was taken over by Taz1p, a protein whose occurrence is limited to *Schizosaccharomyces* and *Pneumocystis* spp. As *S*. *pombe* contains genes encoding all proteins employed as TBPs in yeasts it represents a suitable model for studying the evolution of telomere-binding proteins. The telomeric DNA motifs are from [7,41,48–52]. ^1^Telomeric repeats are heterogeneous in *S*. *cerevisiae*, *S*. *pombe* and *S*. *japonicus*; ^2^(Δ), (Δ-), (-Δ) or (ΔΔ) indicate that corresponding proteins possibly lack DBD, DBD-1, DBD-2 or both DBDs, respectively (see also Table 2). ^3^Tbf-x is an additional Tbf1-like protein encoded by the *P*. *jiroveci* genome. The phylogeny was calculated from concatenated multiple sequence alignments of conserved mitochondrial proteins (i.e. Atp6-8-9-Cob-Cox1-2-3) by the maximum likelihood algorithm and LG (Le-Gascuel) amino acids substitution model implemented in the PhyML program [53]. Bootstrap values (out of 100 replicates) are shown above or below the corresponding branches.

The possibility (iii) of co-evolution of telomeric repeats and TBPs, i.e. expansion of the repertoire of TBPs by duplication and specialization, can be addressed by investigation of telomere protection in yeast species, where Rap1p does not play a role at telomeres (Fig 1). Which proteins act as TBPs in these cases? One potential candidate would be Tbf1p that was shown to bind TTAGGG-like repeats in subtelomeric regions of *S*. *cerevisiae* and is present in most ascomycetes (Fig 1). However, although it can serve as a TBP in *S*. *cerevisiae* with humanized telomeres [19,20], its role at native yeast telomeres is unlikely. Rather, in addition to its binding in subtelomeres, Tbf1p serves as a transcription factor for essential genes such as those encoding ribosomal proteins [21].

*Yarrowia lipolytica* does not contain any putative Rap1p homologue and its telomeres are bound by Tay1 protein [22]. Tay1p contains two Myb domains that mediate its high-affinity binding to *Y*. *lipolytica* telomeric repeats. Remarkably, the affinity of Tay1p to mammalian telomeric repeats is higher than to its native telomeres [6]. This is most likely a result of a functional evolution. Tay1p is a homologue of TBP in the basidiomycete *Ustilago maydis*, which has a mammalian type of telomeric repeats at the ends of its nuclear chromosomes [23]. As in *U*. *maydis*, Tay1p seems to be the only TBP in *Y*. *lipolytica*. In addition, Tay1p probably also acts as a transcription factor for essential genes since the deletion of *TAY1* gene seems to be lethal [22].

Interestingly, *S*. *pombe* and its closely related species contain a Tay1 homologue (Teb1p, also called SpX, Mug152, *Sp*Tay1) [24,25] that retained its function as an essential transcription factor, but apparently lost its role as a TBP, at least in vegetatively growing cells [26]. *S*. *pombe* cells have a Rap1p homologue, but the protein apparently does not bind to DNA [27]. As in other yeast species, the role of *Sp*Tbf1p at telomeres is questionable, although its overproduction leads to a slight increase in the length of telomeric restriction fragments [28]. Instead, fission yeasts feature another TBP called Taz1p [29,30]. Taz1p is similar to TRF1 and TRF2 in having a single Myb domain and binding to telomeres as a homodimer [24,25,31], but it does not seem to be a structural counterpart of the mammalian TBPs [32].

*S*. *pombe* represents an excellent model to investigate the evolutionary path(s) leading to expansion and specialization of TBPs. It exhibits all three proteins (Rap1p, Tay1p/Teb1p and Taz1p) that are employed as TBPs in yeast species belonging to distinct phylogenetic lineages. Based on its position on the phylogenetic tree (Fig 1) we hypothesize that ancestors of *Schizosaccharomyces* spp., just like *Y*. *lipolytica*, employed Tay1p/Teb1p as their TBPs. We speculate that the transition from mammalian to *S*. *pombe* type telomeric repeats was accompanied by the emergence of Taz1p that became specialized for telomeric functions. On the other hand, Teb1p retained functions related to the internal parts of the genome. In this study we use a combination of several quantitative and qualitative approaches to investigate the biochemical properties that might be responsible for diversification of these two proteins in fission yeast.

## Materials and Methods

### Purification of Teb1 protein from *Escherichia coli*

The entire open reading frame (ORF) for Teb1p was amplified from *S*. *pombe* cDNA using primers Teb1_6HN_F and Teb1_6HN_R (S1 Table). The resulting PCR fragment was cloned into pEcoli-Nterm 6xHN vector (Clontech) according to the instructions of the vector supplier resulting in the plasmid p6HN-Teb1 (see S1 Fig for details about construction of expression vectors). As the preliminary experiments indicated that a large fraction of the recombinant protein is present in the insoluble material (data not shown), we fused 6HN-Teb1 coding sequence with a recognition site for *PreScission* protease followed by the ORF for glutathione-*S*-transferase (GST) (S1 Fig). Namely, the 3' end of *teb1*^+^ (233 bp without stop codon) was amplified using the primers fwMug152 and rvMug152nostop (S1 Table) and 1 unit of Phusion High-Fidelity DNA Polymerase (Thermo Scientific). The resulting PCR product was digested with restriction endonucleases *Eco*RI (the restriction site is present within the PCR product) and *Sal*I (the restriction site is provided by the primer rvMug152nostop) (S1 Fig). The sequence for GST and recognition site for PreScission protease were amplified from the plasmid pGEX-6P-1 (GE Healthcate Life Sciences) using the primers fwSalIppsiteGSTstart and rvGSTstopNotI (S1 Table) and 1 unit of Phusion High-Fidelity DNA Polymerase (Thermo Scientific). The resulting PCR product was digested with restriction endonucleases *Not*I and *Sal*I (the restriction sites are provided by the primers). Both digested PCR products were then ligated into the p6HN-Teb1 digested with *Eco*RI and *Not*I as illustrated by S1 Fig. The resulting plasmid (p6HN-Teb1-GST) encoding full-length (with an exception of the first methionine, i.e. amino acids 2–390) tagged Teb1p was verified by DNA sequencing (Microsynth), then transformed into BL21-Gold(DE3)pLysS cells and the transformants were grown on LB plates containing 100 μg/ml ampicillin and 34 μg/ml chloramphenicol. The cells were then inoculated into 30 ml of LB media containing 100 μg/ml ampicillin and 34 μg/ml chloramphenicol and cultivated overnight (15 hours) at 37°C at 225 rpm. The cells were centrifuged for 5 min at 3,000 rpm (Sorvall RT 7 Plus rotor) at 25°C, washed once with LB, inoculated into 1 liter of LB containing 100 μg/ml ampicillin and 34 μg/ml chloramphenicol and cultivated at 37°C at 275 rpm until the *A*_600_ reached a value of 0.6. The culture was cooled to 28°C, followed by addition of isopropyl β-D-1-thiogalactopyranoside (IPTG; final concentration 0.7 mM) and cultivated 1 hour at 28°C and after that 3 days at 17°C. The bacterial cells were then harvested by centrifugation for 15 min at 5,000 rpm at 4°C (F10-6x500y rotor in Sorvall RC 6+), washed once with 200 ml of ice-cold phosphate-buffered saline and the pellet was frozen at -20°C. The pellet was thawed on ice (30–45 min) and resuspended in a final volume of 30 ml of buffer A (20 mM HEPES-NaOH (pH 7.5), 150 mM NaCl, 10 mM 2-mercaptoethanol) containing 1x Complete^™^ (EDTA-free) (Roche), 10 mM MgCl_2_, 100 U of DNase I (Applichem) and 2 μg of PureLink RNase A (Invitrogen). Lysozyme (Sigma-Aldrich) was added to a final concentration of 1 mg/ml and the suspension was incubated for 15 min on ice with occasional shaking. The cells were broken by sonication (5 x 30 sec at a setting of 6 (Branson Sonifier 450)). Each cycle of sonication was followed by 1 min incubation on ice. Triton X-100 was added to a final concentration of 0.1% (v/v). The suspension was sonicated one more time for 30 seconds and incubated for additional 15 min on ice. The insoluble material was pelleted by 30 min centrifugation at 12,000 rpm at 4°C (F21-8x50y in Sorvall RC 6+). The supernatant was mixed with 0.5 ml of a bed volume of glutathione-agarose (Sigma) equilibrated with 1 x 10 volumes of buffer A. The whole suspension was transferred to a 50 ml Falcon tube and incubated for 60–90 min rocking at 4°C. The beads were then washed 1 x 10 volumes of buffer A, followed by addition of PreScission protease (GE Healthcare) in buffer B (50 mM Tris-HCl (pH 7.5), 150 mM NaCl, 1 mM EDTA, 1 mM DTT) and incubated for 2 hours on ice. The beads were then transferred into a column and the cleaved proteins were eluted with 6 washes of 1 ml of buffer B.

### Purification of Taz1 protein from *E*. *coli*

**Fig 2:**
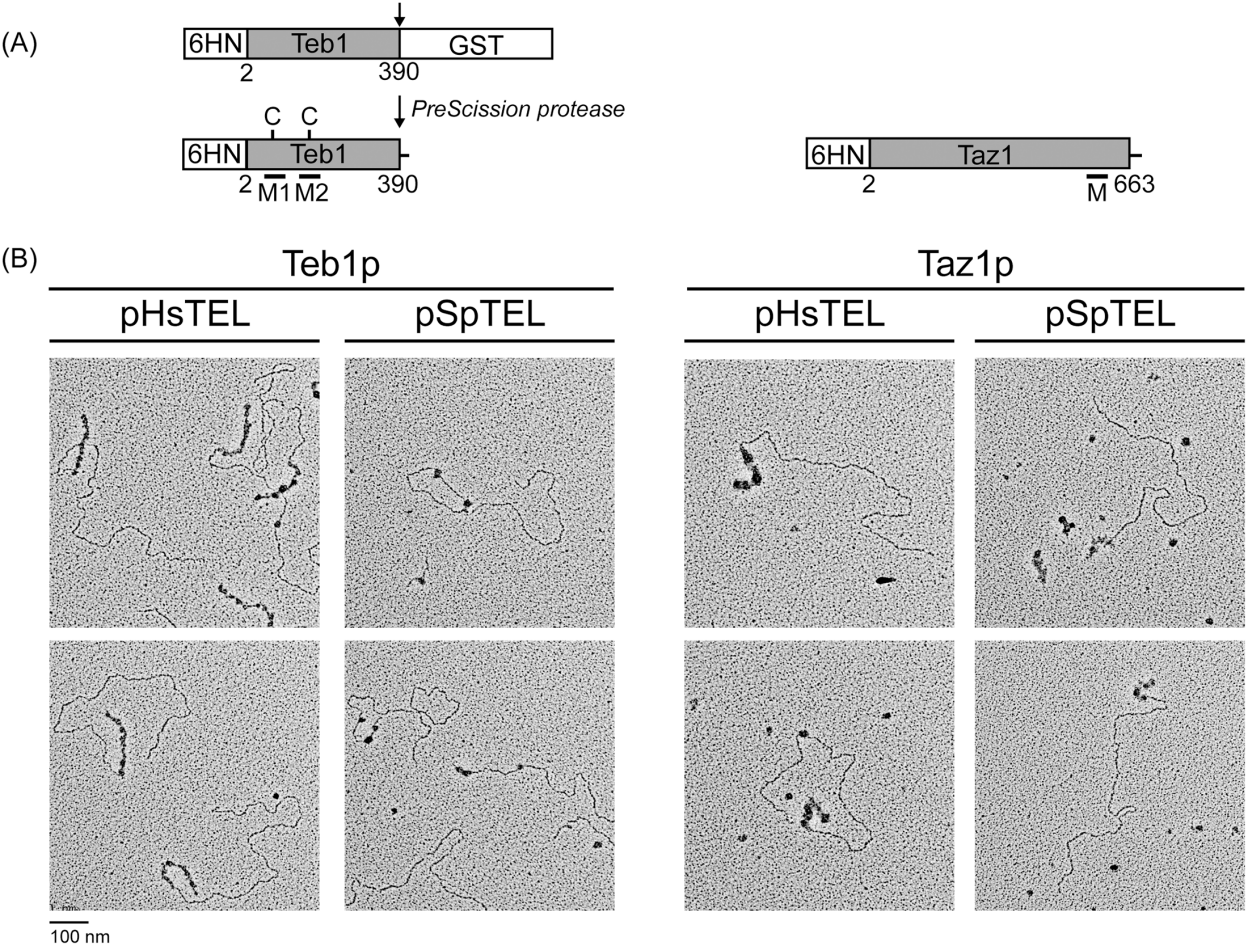
Electron microscopic analysis of the binding of Teb1p and Taz1p to model mammalian and fission yeast telomeres. (A) Schematic representation of the proteins used in all experiments. 6HN-Teb1 was produced in a fusion with GST, purified on glutathione-agarose and GST was removed by cleavage by *PreScission* Protease (the arrow indicates a position of the protease recognition site). Both proteins are represented by their full-length sequences (lacking the first methionine) fused with a 6HN tag at the N-terminus. The numbers below the rectangles indicate the range of amino acids from Teb1p and Taz1p present in the recombinant proteins. C, two cysteine residues located in Myb-1 (M1) and Myb-2 (M2) domain of Teb1p, respectively, that may be involved in mediating inhibitory effect of reducing agents on binding of Teb1p to SpTEL (see below). Note that Taz1p contains a single Myb domain (M) a it carries nine cysteine residues (not shown) and in contrast to Teb1p reducing agent is required for efficient DNA-binding. (B) Linearized plasmids carrying either mammalian (HsTEL) or *S*. *pombe* (SpTEL) telomeres at one end were incubated with purified Teb1p or Taz1p and the DNA-protein complexes were visualized by EM as described in Materials and Methods.

The ORF encoding Taz1p was amplified from genomic DNA of *S*. *pombe* (the *taz1*^+^ gene does not contain any intron) using the primers Taz1_6HN_F and Taz1_6HN_R (S1 Table) and cloned into pEcoli-Nterm 6xHN vector (Clontech) according to the instructions of the vector supplier resulting in the plasmid p6HN-Taz1 (S1 Fig) encoding full-length (with an exception of the first methionine, i.e. amino acids 2–663) Taz1 and containing 6HN affinity tag on N-terminus (Fig 2A). The plasmid was transformed into BL21-Gold(DE3)pLysS cells and the transformants were grown on LB plates containing 100 μg/ml ampicillin and 34 μg/ml chloramphenicol. The cells were then inoculated into 30 ml of LB media containing 100 μg/ml ampicillin and 34 μg/ml chloramphenicol and cultivated overnight (15 hours) at 37°C at 225 rpm. The cells were centrifuged for 5 min at 3,000 rpm (Sorvall RT 7 Plus) at 25°C, washed once with LB, inoculated into 1 liter of LB containing 100 μg/ml ampicillin and 34 μg/ml chloramphenicol and cultivated at 37°C at 275 rpm until the *A*_600_ reached a value of 0.7–0.8. The culture was cooled to 28°C, followed by addition of IPTG (final concentration 1 mM) and cultivation for additional 3 hours at 28°C. The bacterial cells were then harvested by centrifugation for 15 min at 5,000 rpm at 4°C (F10-6x500y rotor in Sorvall RC 6+), washed once with 200 ml of ice-cold phosphate-buffered saline and the pellet was frozen at -20°C. The pellet was thawed on ice (30–45 min) and resuspended in a final volume of 30 ml of buffer B (20 mM HEPES-NaOH (pH 7.5), 300 mM NaCl, 1 mM DTT) containing 1x Complete^™^ (EDTA-free) (Roche), 10 mM MgCl_2_, 100 U of DNase I (Applichem) and 2 μg of PureLink RNase A (Invitrogen). Lysozyme was added to a final concentration of 1 mg/ml and the suspension was incubated for 15 min on ice with occasional shaking. The cells were broken by sonication (5 x 30 sec at a setting of 6 (Branson Sonifier 450)). Each cycle of sonication was followed by 1 min incubation on ice. Triton X-100 was added to a final concentration of 0.1% (v/v). The suspension was sonicated one more time for 30 seconds and incubated for additional 15 min on ice. The insoluble material was pelleted by 30 min centrifugation at 12,000 rpm at 4°C (F21-8x50y in Sorvall RC 6+). The supernatant was mixed with 0.5 ml of a bed volume of the TALON Superflow Metal Affinity Resin (Clontech) equilibrated with 1 x 10 volumes of buffer B. The whole suspension was transferred to a 50 ml Falcon tube and incubated for 60–90 min rocking the tube end-over-end at 4°C. The beads were then washed 1 x 10 volumes of buffer B containing 0.1% (v/v) Triton X-100, followed by 1 x 10 volumes of buffer B. The beads were then transferred into a column and the bound proteins were eluted with 6 washes of 1 ml of elution buffer (20 mM HEPES-NaOH (pH 7.5), 300 mM NaCl, 500 mM imidazole (pH 7.7)).

The fractions containing purified proteins were pooled and loaded onto 5 ml PD MidiTrap G-25 columns (GE Healthcare) pre-washed 3 times with buffer C (50 mM Na-phosphate buffer (pH 7.0), 50 mM NaCl). The presence and purity of the proteins were verified by 10% SDS-PAGE [33] stained with Coomassie Brilliant Blue R-250. Concentrations of proteins were determined by the Bradford assay (Bio-Rad), and proteins were used immediately or stored in 100 μl aliquots at -80°C for longer period.

### Electron microscopy

Plasmid pRST5 carrying about 576 bp of mammalian telomeric repeats [34] was digested with *Bsm*BI and *Not*I resulting in linear DNA fragments containing the repeats at one end of the molecule. Plasmid pSpTEL-NAT was prepared by cloning a ~300 bp segment of the plasmid pNSU70 (kindly provided by Dr. Julie Cooper, Cancer Research UK, London) carrying native *S*. *pombe* telomeric tract [35], see also http://www.pombase.org/status/telomeres) into the pBlueScript II SK vector next to the *Bsm*BI site. Digestion of the plasmid with *Bsm*BI yielded linear DNA fragments containing native *S*. *pombe* telomere at one end. Both plasmids were gel purified using Zymoclean Gel DNA recovery kit (Zymo Research). The Teb1p-DNA-binding reactions for electron microscopy were performed in 50 μl of HNE buffer (20 mM HEPES-NaOH pH 7.5, 1 mM EDTA-NaOH pH 8.0, 100 mM NaCl) containing 2 ng/μl of DNA. The Taz1p-DNA-binding reactions were identical except that they were performed in HNE buffer containing 0.1 mM DTT (HNED). The reactions were carried out at room temperature for 15 min, followed by addition of 10 μl of 1.2% (v/v) glutaraldehyde and incubation at room temperature for additional 6 min. To remove the unbound proteins and fixative, the samples were diluted to 50 μl in HNE buffer and passed over 2 ml columns of 6% agarose beads (ABT Inc., Burgos, Spain) equilibrated with TE buffer (10 mM Tris-HCl, pH 7.4, 0.1 mM EDTA-NaOH). Aliquots of the fractions containing the complexes were mixed with a buffer containing spermidine and adsorbed onto copper grids coated with a thin carbon film glow-charged shortly before sample application. Following adsorption of the samples for 3 min, the grids were dehydrated through a graded ethanol series and rotary shadowcast with tungsten at 10^−7^ torr [36] Samples were examined in an FEI T12 TEM equipped with a Gatan 2kx2k SC200 CCD camera.

### DNA substrates and electrophoretic-mobility shift assay (EMSA)

For EMSA, oligonucleotides SpTEL_A, SpTEL-M1_A, SpTEL-M2_A, SpTEL-M3_A, HsTEL_A, HisBox_A, HisBoxMut_A, HisBoxFlank_A, HisBoxFlank3'_A, HisBoxFlank5'_A, HisBoxFlankPart_A, respectively (S1 Table), were radioactively labeled using T4 polynucleotide kinase (Life Technologies) and [γ^32^P]ATP. The labeled oligonucleotides were then mixed with non-labeled complementary oligonucleotides (S1 Table) in a molar ratio 1:3. The mixtures were incubated at 95°C for 5 min and cooled slowly to room temperature to allow DNA annealing. The unincorporated [γ^32^P]ATP was removed from the DNA by gel filtration using Probe Quant G-50 MicroColumns (GE Healthcare). Purified recombinant proteins at concentrations from 0.05 to 4.5 μM were mixed with the corresponding DNA substrate (15 nM) and incubated for 10 min at room temperature in 10 μl HNE (Teb1) or HNED (Taz1) buffer. Samples were electrophoretically separated in 5% (v/v) polyacrylamide gels in 0.5x TBE buffer (45 mM Tris-borate, 1 mM EDTA-NaOH pH 8.0). DNA and DNA-protein complexes were visualized after exposing the gels to storage phosphor screens (Kodak) for 24–72 hours using Personal molecular imager FX (BioRad).

### Fluorescence anisotropy

The equilibrium binding of Teb1 and Taz1 proteins variants to DNA oligonucleotide duplexes was analyzed by fluorescence anisotropy. Measurements were carried out with slight modifications to a previously described protocol [37]. The corresponding DNA oligonucleotide labeled with the FAM at 5’-end was allowed to hybridize with the complementary oligonucleotide at an equimolar ratio. The complete formation of the duplex was verified by PAGE. The DNA oligonucleotides were supplied by VBC Biotech (Vienna, Austria). The measurements of fluorescence anisotropy were conducted on a FluoroMax-4 spectrofluorometer (Horiba Jobin-Yvon, Edison, NJ) equipped with a thermostable cell holder and magnetic stirrer. Samples were excited with vertically polarized light at 494 nm and both vertical and horizontal emissions were recorded at 520 nm. The integration time was 1 s; dsDNA probes (2 nM) were titrated at 25°C with Taz1 (8 μM) in HNED buffer. Fluorescence anisotropy measurements of binding of Teb1 to dsDNA probes were the same as for Taz1p except that the experiments were carried out using the HNE buffer. Teb1 binding to SpTEL was measured at 37°C, HsTEL and HisBox were titrated at 25°C. A fixed delay of 120 s was set between each protein aliquot addition and start of the measurement to allow the binding reaction to reach equilibrium. This delay was sufficient, as no further change in anisotropy was observed. Each data point is an average of three measurements. The experimental binding isotherms were analyzed by non-linear least squares regression in SigmaPlot 11 software (Systat Software) using a single-site binding model according to [38] and confirmed by numerical approach using DynaFit software [39].

## Results

Two earlier reports provided important yet only partial characterization of Teb1p and Taz1p binding to DNA [24,25]. The main limitation of these studies is that they employed *in vitro* translated versions of the proteins or crude protein extracts, thus precluding precise quantification of DNA binding. In addition, it was not possible to exclude interference of other proteins present in the extracts with the DNA binding. Furthermore, while the earlier study [25] indicated that Teb1p binds to *S*. *pombe* telomeres *in vitro* (although with lower affinity than to mammalian telomeres), Spink et al. [24] reported that Teb1p does not bind to the fission yeast telomeric probe at all. To resolve these conflicting issues and to obtain a detailed view on the DNA-binding properties of Teb1p and Taz1p, we purified recombinant full-length versions of the proteins and assessed their binding to a battery of DNA substrates using three different approaches, namely electron microscopy (EM), electrophoretic mobility shift assays (EMSA) and fluorescence anisotropy (FA).

### Electron microscopy visualization of Teb1p and Taz1p binding to model mammalian and *S*. *pombe* telomeres

As a mammalian telomere model we used the plasmid pRST5 carrying a ~576 bp array of TTAGGG repeats [34]. We also constructed an analogous plasmid carrying ~300 bp of native *S*. *pombe* telomere sequence [35] into pBlueScript SK(+) resulting in the vector pSpTEL-NAT. Both pRST5 (further HsTEL) and pSpTEL-NAT (SpTEL) were linearized to place the corresponding telomeric tract at one end of the molecules and incubated with either Teb1p or Taz1p. The reaction conditions were identical except that the buffer for Taz1p contained 0.1 mM DTT that was essential for Taz1p binding, but it inhibited Teb1p binding to DNA. The DNA-protein complexes were purified by gel-filtration and visualized by EM (Fig 2). Under the reaction conditions employed, the binding of Teb1p to HsTEL was very similar with that exhibited by *Y*. *lipolytica* Tay1p [6,22]. Taz1p binding to HsTEL is also restricted to the terminal telomeric tract, although compared with Teb1p, the binding is more scattered along the tract and the particles are much larger. Much more dramatic differences were observed when SpTEL was used as a substrate. Whereas there were 1–3 Teb1 protein particles per telomeric tract (and sometimes within the nontelomeric region of the plasmid), Taz1p formed an array of particles along the terminal region of the plasmid. In conclusion, the EM experiments indicate that whereas Taz1p binds similarly to both HsTEL and SpTEL, the ability of Teb1p to bind SpTEL is rather weak, while its binding to HsTEL suggests a preference for TTAGGG repeats.

### Electrophoretic mobility shift assays and fluorescence anisotropy reveal the differences in requirements of Teb1p and Taz1p for DNA binding

It is important to note that the cloned native telomere [35] used for construction of SpTEL-NAT contains no TTAGGG repeat and the occurrence of mammalian type telomeric repeat in *S*. *pombe* telomeres is less than 1% [40]. On the other hand, TTAGGG repeats are present in internal parts of *S*. *pombe* chromosomes including regulatory regions 5’ upstream of several protein-coding genes. Of special interest are promoters of genes encoding canonical histones, each carrying the sequence AGGGTTAGGGTT(t/g)tgat (further HisBox), where the underlined sequence corresponds to two mammalian telomeric repeats. The whole genome ChIP-chip experiments indicated that the HisBox mediates binding of Teb1p to all promoters of canonical histones *in vivo* [26]. Thus, we could compare the preferences of Teb1p and Taz1p to HsTEL (the substrate recognized by both proteins), SpTEL (*in vivo* substrate for Taz1p) and HisBox (*in vivo* substrate for Teb1p).

First, we optimized the conditions for binding of both proteins to DNA. As indicated above, the presence of a reducing agent (DTT or 2-mercaptoethanol) was required for binding of Taz1p, whereas this was inhibitory for the DNA binding activity of Teb1p to SpTEL although the affinity to HsTEL and HisBox was only marginally affected (data not shown). Teb1p contains two cysteine residues (Cys-70 and Cys-161) and it is possible that they may be involved either in dimerization of the protein or they may directly participate in DNA binding to non-preferred substrates (such as SpTEL) as they are located within the Myb-1 and Myb-2 domain, respectively (Fig 2A). We also tried to replace sodium chloride with lithium chloride to destroy potential secondary DNA structures, but we did not observe any difference in binding (data not shown). Based on these results we employed HNE buffer for Teb1p and HNED buffer for Taz1p (see Materials and Methods) in both EMSA and fluorescence anisotropy experiments.

**Fig 3:**
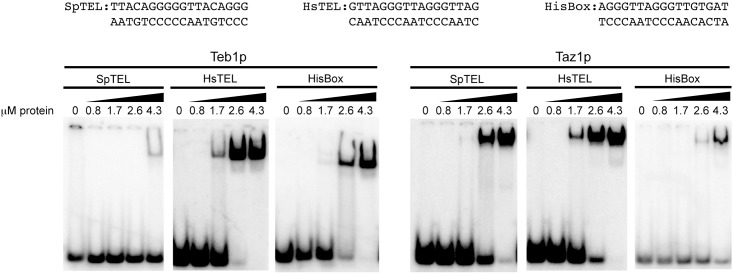
Comparison of the binding of Teb1p and Taz1p to mammalian telomeres, fission yeast telomeres and HisBox using EMSA. The indicated radioactively labeled DNA probes were incubated with increasing concentrations (indicated above the lanes) of Teb1p or Taz1p and DNA (15 nM) and DNA-protein complexes were separated by electrophoresis in 5% polyacrylamide gels as described in Materials and Methods.

When we used HsTEL and SpTEL DNA probes in EMSA, the results corroborated the EM data. Both proteins bound equally well to HsTEL. On the other hand, whereas Taz1p bound strongly to SpTEL, Teb1p exhibited significantly weaker binding to this substrate (Fig 3). Again, as in the case of the native telomere, the SpTEL probe does not contain a TTAGGG motif, which seems to facilitate Teb1p binding to DNA. To test this hypothesis, we employed a HisBox probe containing two mammalian telomeric repeats (see above). Although this DNA sequence was bound by both proteins, it is clear that Teb1p starts shifting the DNA probe at lower concentrations indicating that it exhibits a higher affinity for this substrate.

**Fig 4:**
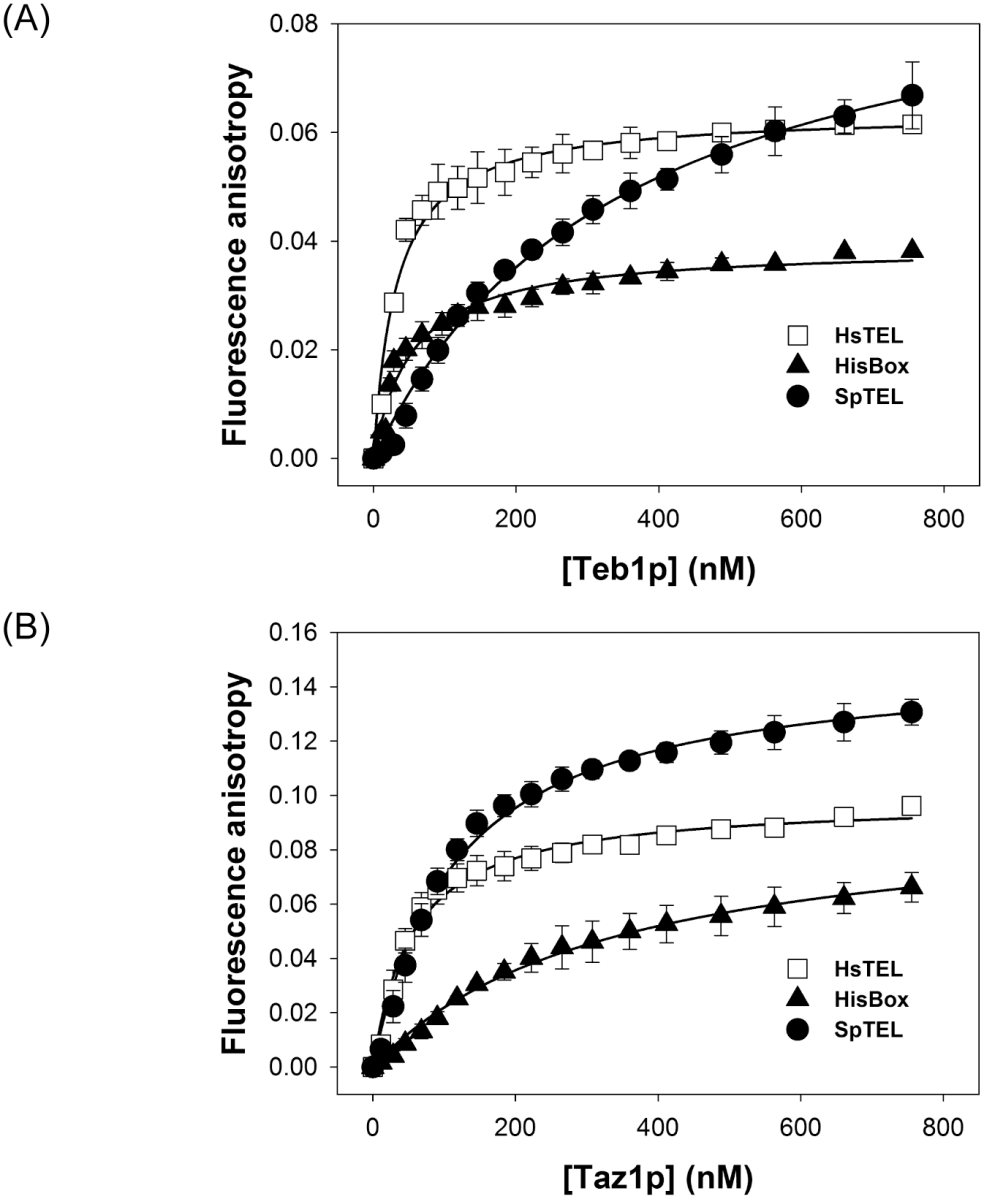
Fluorescence anisotropy analysis of the DNA binding properties of Teb1p and Taz1p. Fluorescence anisotropy measurements of binding of Teb1p (A) and Taz1p (B) to FAM labeled DNA probes were performed as described in Materials and Methods. SpTEL (closed circle), HsTEL (open square), HisBox (closed triangle).

To assess the DNA-binding properties of Teb1p and Taz1p quantitatively, we measured dissociation constants (*K*_*D*_) corresponding to Teb1p or Taz1p binding to HsTEL, SpTEL and HisBox by fluorescence anisotropy (FA) (Fig 4 and Table 1). If the solution contains only free fluorescently labeled DNA molecules, the FA is relatively low, owing to the fast rotational rearrangement of DNA molecules. If the protein aliquots are added to the solution of labeled DNA, a bulky slower-rotating protein—DNA complex is formed and the anisotropy value increases. We used HsTEL, SpTEL and HisBox (labeled with FAM) as DNA substrates for Teb1p and Taz1p binding assays. The anisotropy change described the extent of Teb1p and Taz1p binding to telomeric DNA duplex. The equilibrium isotherms were recorded and binding affinity was quantified by a fitting analysis (see Materials and Methods for details).

**Table 1:** Parameters of binding of Teb1 and Taz1 proteins to *S*. *pombe* telomere (SpTEL), mammalian telomeres (HsTEL) and *S*. *pombe* histone box (HisBox).

	SpTEL		HsTEL		HisBox	
	*K*_*D*_ [nM]	*K*_*a*_ [10^6^M^-1^]	*K*_*D*_ [nM]	*K*_*a*_ [10^6^M^-1^]	*K*_*D*_ [nM]	*K*_*a*_ [10^6^M^-1^]
**Teb1**	380 ± 30	2.6 ± 0.2	31 ± 5	34 ± 5	53 ± 6	20 ± 2
**Taz1**	115 ± 9	8.7 ± 0.7	54 ± 5	19 ± 2	380 ± 50	2.6 ± 0.4

Importantly, both the equilibrium association (*K*_*a*_) and dissociation (*K*_*D*_) constants obtained for DNA binding of Teb1p and Taz1p (Table 1) agreed with the results of the EMSA experiments. Both proteins exhibited the highest affinity to HsTEL. The affinities for the other two DNA substrates differed. Whereas Taz1p bound relatively strongly to SpTEL, Teb1p exhibited only a weak binding to this substrate and the pattern was reversed with the HisBox probe.

### Teb1p requires the presence of two mammalian type telomeric repeats for efficient binding, whereas Taz1p seems to be a more flexible DNA-binding protein

**Fig 5:**
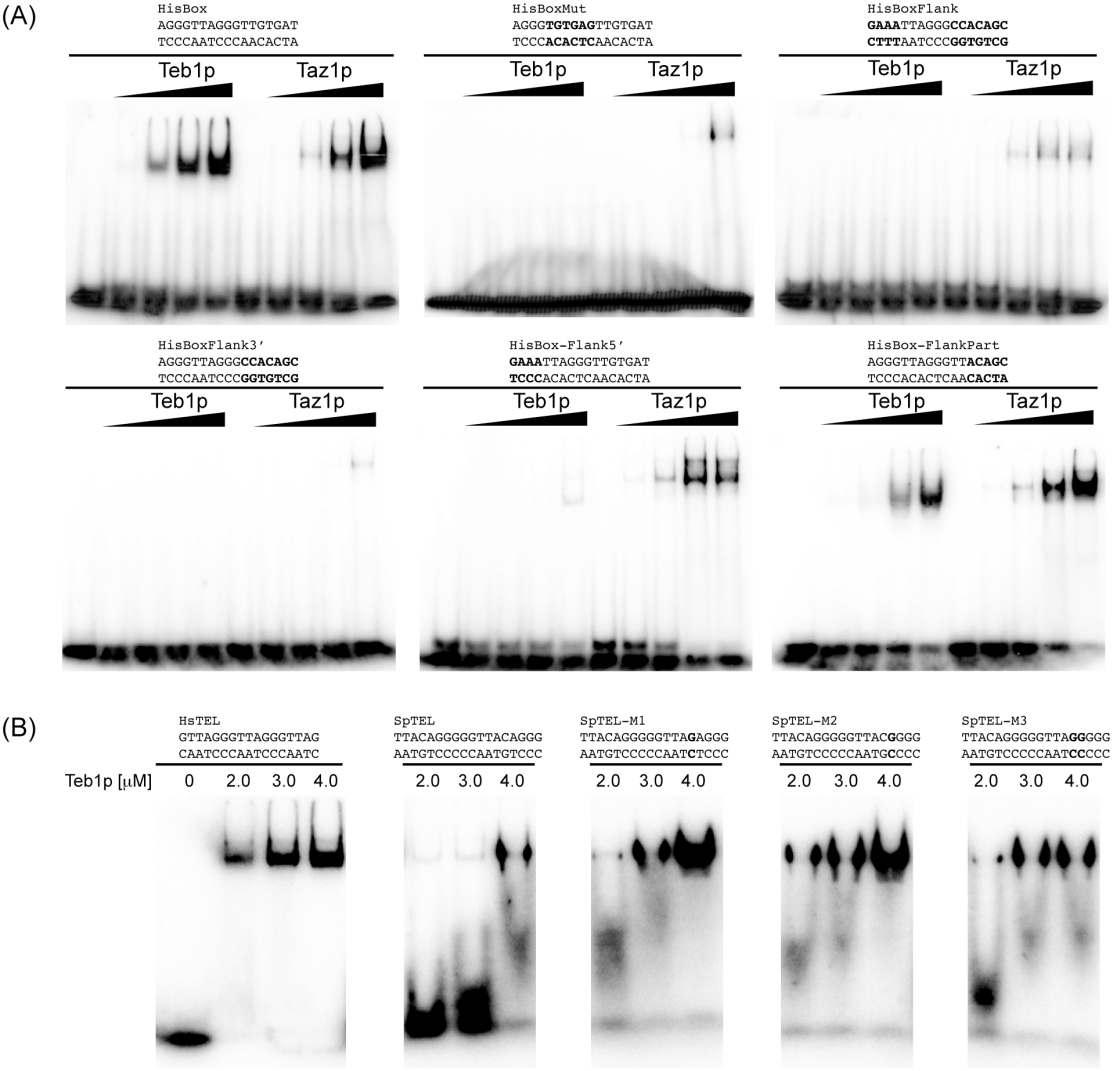
Teb1p binds effectively to a narrow range of DNA sequences. (A) Teb1p and Taz1p exhibit different requirements for binding to HisBox. Concentrations of the proteins are as in Fig 3. (B) Modified versions of SpTEL as substrates for Teb1p. The indicated radioactively labeled DNA probes were incubated with indicated concentrations of the protein and DNA (15 nM) and DNA-protein complexes were separated by electrophoresis in 5% polyacrylamide gels as described in Materials and Methods. Substituted nucleotides are highlighted in bold.

The fact that both Teb1p and Taz1p bind HisBox (although with different affinities) enabled us to define the nucleotide sequence requirements of the two proteins to this DNA substrate. To this end we tested five different variants of HisBox (Fig 5A). First, we replaced the central TTAGGG sequence by its scrambled version (TGTGAG; HisBoxMut). This change resulted in a complete loss of binding of Teb1p, while Taz1p was able to bind the probe only at the highest concentration. Interesting differences were observed when the central TTAGGG sequence was retained and the 5' or 3' flanking sequences were mutated. Whereas for Teb1p the flanking sequences at both the 5' and 3' sides of the TTAGGG were needed for efficient binding, Taz1p required that only the 3' flanking sequence be preserved. Almost complete restoration of Teb1p binding was achieved when the 3' flanking sequence contained a TT sequence indicating that the minimal Teb1p recognition sequence seems to correspond to two mammalian type telomeric repeats (configured as 5'-AGGGTTAGGGTT-3').

We also tested for the minimal changes in SpTEL that would result in a more efficient binding of Teb1p. We have prepared three different versions of SpTEL (M1-M3) that instead of the sequence 5'-TTACAG-3' contain a repeat unit nearly identical (5'-TTAGAG-3' in M1 and 5'-TTACGG-3' in M2), or identical (5'-TTAGGG-3' in M3) to mammalian telomeric repeat (Fig 5B). In agreement with the results presented above (Fig 3), unlike the HsTEL probe, SpTEL sequence is a poor substrate for Teb1p. Interestingly, a single substitution within the target sequence (M1, M2) resulted in a more efficient binding of the protein. However, the DNA-protein complex seems to be unstable as judged by its migration during electrophoresis (Fig 5B). This indicates that to achieve efficient and tight binding Teb1p is relatively strict in its requirements for the target DNA sequence.

## Discussion

In this study we employed purified recombinant Taz1p and Teb1p in three types of DNA-binding assays (EMSA, fluorescence anisotropy, electron microscopy) and quantitatively assessed their DNA-binding properties thus enabling their comparative biochemical analysis. Our results confirm that both Teb1p and Taz1p exhibit relatively high affinity to mammalian type telomeres, but only Taz1p binds well the fission yeast native telomere (Fig 3) [24,25]. Our data (Fig 4) indicate that Teb1p is more strict in its requirements for TTAGGG-like repeats, which is illustrated by the results of the EMSA experiments with variants of the HisBox and SpTEL probes (Fig 5). It seems that similar to *S*. *cerevisiae* Rap1p [15–18], Taz1p is more flexible in terms of recognition of the target DNA. This is most likely caused by the relatively large degree of heterogeneity of *S*. *pombe* telomeric repeats, differing in both length and sequence. This flexibility is probably also responsible for the fact that Taz1p binds to HisBox and exhibits less strict requirements for the regions flanking the central TTAGGG sequence (Fig 5A).

The stringency of Teb1p binding to TTAGGG-like sequences may be the main reason for its replacement by Taz1p in fission yeast. *Y*. *lipolytica* telomeric repeats (5'-GGGTTAGTCA-3') also differ from the canonical TTAGGG and Tay1p exhibits about 5-fold lower affinity to its native telomeres than to mammalian type repeats [6]. However, the DNA-binding properties of Tay1p seem to be still suitable for performing the functions as a TBP. On the other hand, the fact that the occurrence of TTAGGG repeats is extremely rare in *S*. *pombe* may have been one of the driving forces for replacement of Teb1p by the more flexible Taz1p. Recently, it was shown that Taz1p is not a structural counterpart of mammalian TRF1 and TRF2 [32]. It is possible that the evolution of complex telomeric repeats characteristic for *Schizosaccharomyces* spp. such as *S*. *pombe*, *S*. *cryophilus*, *S*. *japonicus* or *S*. *octosporus* (Fig 1, [41]) was made possible by the *de novo* origin of the Taz1p-encoding gene as a result of a recruitment of a Myb domain by a protein that was able to form homodimers. Such a gene could have been already present in the ancestral lineage as *Pneumocystis jiroveci* seems to contain a Taz1p homologue, although it is considerably shorter than in *Schizosaccharomyces* spp. (Fig 1 and Table 2). The distribution of various TBPs on the phylogenetic tree also supports our hypothesis [6] that the ancestral genomes accumulated precursors of various types of TBPs possibly via the neutral evolutionary ratchet [42] involving gene and domain duplications followed by their specialization (or loss) in distinct lineages [43].

**Table 2:** List of putative telomere-binding proteins identified by *in silico* analysis of the corresponding fungal genomes (see also Fig 1).

**Rap1 homologues**						
Species	Uniprot ID	Size [aa]	Identified domains (N-to-C)			
			BRCT^1^	DBD-1^2^	DBD-2^2^	RCT^3^
*C*. *albicans*	Q59XX8	429	+	+	+	-
*C*. *glabrata*	Q96WQ7	687	+	+	+	+
*C*. *parapsilosis*	G8BJX2	1071	+	+	+	+
*K*. *lactis*	Q01073	666	+	+	+	+
*P*. *jiroveci*	L0P7Z4^4^	409	-	-	-	+
*S*. *cerevisiae*	P11938	827	+	+	+	+
*Sch*. *cryophilus*	S9XJW4	708	+	(+)^5^	-	+
*Sch*. *japonicus*	B6K3A8	727	+	+^5^	-	+
*Sch*. *octosporus*	S9PYB2	709	+	(+)^5^	-	+
*Sch*. *pombe*	Q96TL7	693	+	(+)^5^	+	+
*T*. *deformans*	R4XAV8	883	+	+	+	+
**Taz1 homologues**						
Species	Uniprot ID	Size [aa]	Identified domains (N-to-C)			
			TRFH^6^		DBD^2^	
*P*. *jiroveci*	L0PB74^4^	358	-		+	
*Sch*. *cryophilus*	S9XI76	644	+		+	
*Sch*. *japonicus*	B6JYV6	681	-		+	
*Sch*. *octosporus*	S9QYS6	648	(+)		+	
*Sch*. *pombe*	P79005	663	+		+	
**Tbf1 homologues**						
Species	Uniprot ID	Size [aa]	Identified domains (N-to-C)			
			TRFH^6^		DBD^2^	
*C*. *albicans*	Q5AHJ5	886	+		+	
*C*. *glabrata*	Q6FJX7	525	+		+	
*C*. *parapsilosis*	G8BDW4	735	+		+	
*K*. *lactis*	Q6CRS7	473	+		+	
*P*. *jiroveci*	L0PAE7	132	-		+	
*P*. *jiroveci* (Tbf-x)	L0PE60	706	-		+	
*S*. *cerevisiae*	Q02457	562	+		+	
*Sch*. *cryophilus*	S9W2U6	484	+		+	
*Sch*. *japonicus*	B6K1V2	479	+		+	
*Sch*. *octosporus*	S9RER3	484	+		+	
*Sch*. *pombe*	Q6E434	485	+		+	
*U*. *maydis*	A0A0D1E1Z3	1528	-		(+)	
*Y*. *lipolytica*	Q6FJX7	710	+		+	
**Teb1-Tay1 homologues**						
Species	Uniprot ID	Size [aa]	Identified domains (N-to-C)			
			DBD-1^2^		DBD-2^2^	
*P*. *jiroveci*	L0PER7	367	+		+	
*Sch*. *cryophilus*	S9X458	397	+		+	
*Sch*. *japonicus*	B6JZ90	418	+		+	
*Sch*. *octosporus*	S9PN52	397	+		+	
*Sch*. *pombe*	Q10274	390	+		+	
*T*. *deformans*	R4X928	329	+		+	
*U*. *maydis*	A0A0D1CTG7	1127	+		+	
*Y*. *lipolytica*	Q6C9I6	406	+		+	

^1^ BRCT—breast cancer susceptibility protein C-terminal domain

^2^ DBD—DNA-binding domain; DBDs predicted with a low confidence are shown in parentheses

^3^ RCT—Rap1 C-terminal domain (protein interacting domain)

^4^ note that *Sch*. *pombe* Rap1 (and possibly its orthologues from other *Schizosaccharomyces* spp.) does not bind telomeric DNA, but it associates with telomeres via protein-protein interactions (Kanoh and Ishikawa, 2001).

^5^ only partial sequence is available in the UniProt database; note that *P*. *murina* Rap1 homologue possesses a BRCT domain

^6^ TRFH—Telomere repeat-binding factor homology (dimerisation domain)

Naturally, there is still the possibility that Teb1p might retain some of its functions at telomeres, although its biochemical properties *in vitro* do not support this hypothesis ([24,25] and this study). In addition, ChIP-chip data yielded no enrichment of telomeric DNA in Teb1p-immunoprecipitates [26]. Furthermore, temperature-sensitive mutants in the *teb1*^+^ gene exhibited no changes in telomere length [26]. On the other hand, Teb1p might complement or substitute for Taz1p under special circumstances. We investigated (by both EMSA and FA) whether Teb1p interferes with the binding of Taz1p to SpTEL, but we observed no detectable effect (data not shown). Another possibility stems from the observation that the *teb1*^+^ gene is up-regulated during meiosis [44], when telomeres undergo relatively dramatic changes [45–47]. Finally, *taz1*^-^ mutants lacking Taz1p exhibit extremely long and heterogeneous telomeres [29] and the protein composition of telomeric chromatin in these cells is not known. We have shown that Teb1p can, although weakly, bind to native *S*. *pombe* telomeres. It is possible that when provided with a 10-fold increase in the number of telomeric repeats, Teb1p could recognize telomeres in *taz1*^-^ mutants. When we combined the temperature-sensitive *teb1* mutation [26] with *taz1*^-^, the double mutants exhibited telomeres indistinguishable from the single *taz1*^-^ mutant indicating that Teb1p does not play a major role at telomeres in *taz1*^-^ cells (data not shown). However, we have not yet analyzed telomeres by means other than measuring lengths of telomeric restriction fragments, so this possibility is still not fully excluded.

Even if we did not identify telomeric functions for Teb1p, the main significance of this study is that it provides a very useful platform for investigation of evolutionary paths leading to expansion of the repertoire of TBP in yeasts. If we assume that the ancestor of *S*. *pombe* contained TTAGGG-like repeats and Tay1p/Teb1p as the TBP, we could try to reconstruct the evolutionary path leading to division of labor between flexible Taz1p and stringent Teb1p fulfilling their roles at telomeres and in internal parts of the genome, respectively.

## Supporting Information

S1 Fig Scheme for construction of vectors used to express (A) Teb1p and (B) Taz1p in *Escherichia coli*. For detailed description see Materials and Methods. Sequences of the primers used for PCR are listed in S1 Table.
(PDF)

S1 Table List of oligonucleotides. (PDF)
