# Supplementary material for: Evolution of Telomeres in Schizosaccharomyces pombe and Its Possible Relationship to the Diversification of Telomere Binding Proteins
Source: PLoS One. 2016 Apr 21;11(4):e0154225. doi: 10.1371/journal.pone.0154225 (PMC4839565; doi:10.1371/journal.pone.0154225)
Supplement: S1 Fig — For detailed description see Materials and Methods. Sequences of the primers used for PCR are listed in S1 Table. (PDF) [file pone.0154225.s001.pdf]

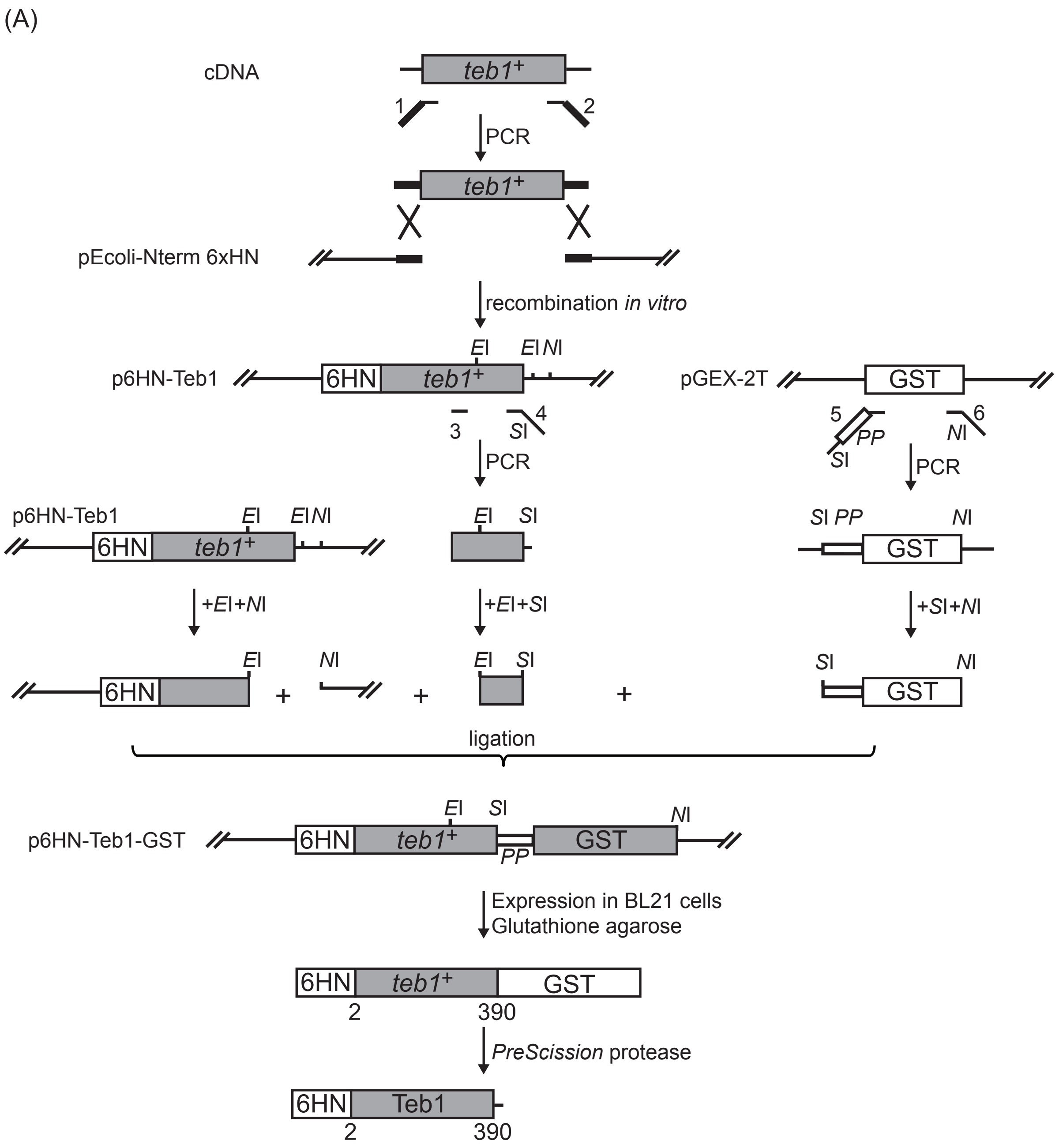

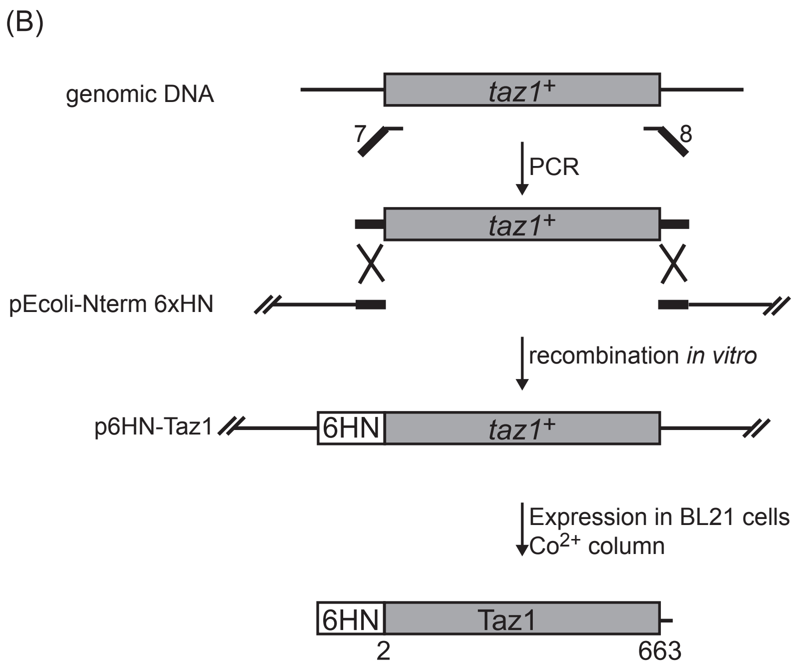

**Primers used for PCR:**	**Abbreviations:**
1, Teb1_6HN_F	6HN, afinity tag
2, Teb1_6HN_R	*E*I, *Eco*RI
3, fwMug152	*N*I, *Not*I
4, rvMug152nostop	*S*I, *Sal*I
5, fwSalIppsiteGSTstart	*PP*, *PreScission* protease
6, rvGSTstopNotI	GST, glutathione-*S*-transferase
7, Taz1_6HN_F	
8, Taz1_6HN_R	

S1 Figure. Scheme for construction of vectors used to express (A) Teb1p and (B) Taz1p in *Escherichia coli*. For detailed description see Material and Methods. Sequences of the primers used for PCR are listed in S1 Table.
