# Supplementary material for: Evolution of Telomeres in Schizosaccharomyces pombe and Its Possible Relationship to the Diversification of Telomere Binding Proteins
Source: PLoS One. 2016 Apr 21;11(4):e0154225. doi: 10.1371/journal.pone.0154225 (PMC4839565; doi:10.1371/journal.pone.0154225)
Supplement: S1 Table — (PDF) [file pone.0154225.s002.pdf]

S1 Table List of oligonucleotides

Name	sequence 5´→3´	Application	Notes
Teb1_6HN_F	*TAAGGCCTCTGTCGACA*GGTCTATGAAACCTCCAGGGT	*1*	italics: sequence derived from the target plasmid pEcoli-NTerm 6xHN<br>underlined: start of the Teb1 ORF excluding the first ATG codon
Teb1_6HN_R	*CAGAATTCGCAAGCTT*CTATCCCCGGTTGTCCCACGGT	*1*	italics: sequence derived from the target plasmid pEcoli-NTerm 6xHN<br>underlined, sequence from 3' end of Teb1 ORF
fwMug152	GCCGCTGCAGGCCAACCTCCTTCATTA	*1*	
rvMug152nostop	ATTAGTCGACTCCCCGGTTGTCCCACGGTAT	*1*	underlined: *Sal*I site
fwSalIppsiteGSTstart	TAATGTCGAC*CTGGAAGTTCTGTTCCAGGGGCCC*ATGTCCCCTATACTAGGTTATT	*1*	underlined: *Sal*I site<br>italics: sequence encoding *PreScission* protease cleavage site
rvGSTstopNotI	TAATGCGGCCGCTCAATCCGATTTTGGAGGATGGTCGC	*1*	underlined: *Not*I site
Taz1_6HN_F	*TAAGGCCTCTGTCGAC*ATAAGCGTGCAAAGTACAGAAACG	*2*	italics: sequence derived from the target plasmid pEcoli-NTerm 6xHN<br>underlined: start of the Taz1 ORF excluding the first ATG codon
Taz1_6HN_R	*CAGAATTCGCAAGCTT*AGATTGATAATTAACAAGCTCTTC	*2*	italics: sequence derived from the target plasmid pEcoli-NTerm 6xHN<br>underlined, sequence from 3' end of Taz1 ORF
HsTEL_S	GTTAGGGTTAGGGTTAG	*3,4*	
HsTEL_A	CTAACCCTAACCCTAAC	*3,4*	
SpTEL_S	TTACAGGGGGTTACAGGG	*3,4*	
SpTEL_A	CCCTGTAACCCCCTGTAA	*3,4*	
HisBox_S	AGGGTTAGGGTTGTGAT	*3,4*	
HisBox_A	ATCACAACCCTAACCCT	*3,4*	
HisBoxMut_S	AGGGTGTGAGTTGTGAT	*3*	underlined: sequence different from the wild-type HisBox
HisBoxMut_A	ATCACAACTCACACCCT	*3*	underlined: sequence different from the wild-type HisBox
HisBoxFlank_S	GAAATTAGGGCCACAGC	*3*	underlined: sequence different from the wild-type HisBox
HisBoxFlank_A	GCTGTGGCCCTAATTTC	*3*	underlined: sequence different from the wild-type HisBox
HisBoxFlank 3´_S	AGGGTTAGGGCCACAGC	*3*	underlined: sequence different from the wild-type HisBox
HisBoxFlank 3´_A	GCTGTGGCCCTAACCCT	*3*	underlined: sequence different from the wild-type HisBox
HisBoxFlank 5´_S	GAAATTAGGGTTGTGAT	*3*	underlined: sequence different from the wild-type HisBox
HisBoxFlank 5´_A	ATCACAACCCTAATTTC	*3*	underlined: sequence different from the wild-type HisBox
HisBoxFlank Part_S	AGGGTTAGGGTTACAGC	*3*	underlined: sequence different from the wild-type HisBox
HisBoxFlank Part_A	GCTGTAACCCTAACCCT	*3*	underlined: sequence different from the wild-type HisBox

Supplementary table 1 List of oligonucleotides (*continued*)

Name	sequence 5´→3´	Application	Notes
SpTEL-M1_S	TTACAGGGGGTTAGAGGG	*3*	underlined: sequence different from SpTEL
SpTEL-M1_A	CCCTCTAACCCCCTGTAA	*3*	underlined: sequence different from SpTEL
SpTEL-M2_S	TTACAGGGGGTTACGGGG	*3*	underlined: sequence different from SpTEL
SpTEL-M2_A	CCCCGTAACCCCCTGTAA	*3*	underlined: sequence different from SpTEL
SpTEL-M3_S	TTACAGGGGGTTAGGGGG	*3*	underlined: sequence different from SpTEL
SpTEL-M3_A	CCCCCTAACCCCCTGTAA	*3*	underlined: sequence different from SpTEL

1 Preparation of the plasmid p6HN-Teb1-GST
2 Preparation of the plasmid p6HN-Taz1
3 EMSA experiments
4 Fluorescence anisotropy analysis
